# Correlation between impostor syndrome among doctoral students and supervisor empathy in Tunisia

**DOI:** 10.3389/fpsyg.2024.1382969

**Published:** 2024-07-29

**Authors:** Oumayma Slimi, Antonella Muscella, Santo Marsigliante, Mourad Bahloul

**Affiliations:** ^1^High Institute of Sport and Physical Education, University of Sfax, Sfax, Tunisia; ^2^Department of Biological and Environmental Sciences and Technologies (Di.S.Te.B.A.), University of Salento, Lecce, Italy; ^3^Higher Institute of Education and Continuing Training, Virtual University of Tunis, Tunis, Tunisia

**Keywords:** impostor syndrome, empathy, doctoral students, socio-professional factors, academic environment, doctoral supervisor

## Abstract

**Introduction:**

The prevalence of impostor syndrome among PhD students has increased rapidly in recent years, having very negative effects on their mental and psychological health as well as on their doctoral journey. This exploratory study aims to examine whether there is a causal correlation between impostor syndrome among PhD students and empathy among supervisors.

**Methods:**

This study encompasses 562 doctoral students (300 females, 262 males) and 152 Tunisian supervisors (68 females, 84 males).

**Results:**

Employing ANOVA, significant influences on impostor syndrome emerge for gender, marital status, professional status, and the doctoral enrollment level (*p* < 0.001). Concurrently, supervisors’ empathy is significantly affected by gender, marital status, and experience (*p* < 0.001). Linear regression establishes a noteworthy negative correlation (*p* = 0.045): a 1-unit increase in empathy correlates with a 0.122-unit decrease in impostor syndrome.

**Discussion:**

These findings underscore the intricate relationship between socio-professional factors, empathy, and impostor syndrome within the academic milieu, offering crucial insights for interventions and psychological support. The study aligns with the broader context of understanding mental health challenges in academia, emphasizing the imperative for ongoing support initiatives.

## 1 Introduction

In the face of escalating global competition in academic research (Kosmützky and Krücken, 2023), pursuing a doctoral thesis presents a formidable challenge for many students, leading to a significant number prematurely abandoning their studies (Bai et al., 2023). Despite their objective successes, some individuals struggle to accept their achievements and fear being perceived as impostors. This phenomenon, often referred to as the “impostor syndrome” (Clance and Imes, 1978; Harvey, 1981; Bravata et al., 2020), has been extensively studied for its negative effects on individuals’ well-being (Sonnak and Towell, 2001) as well as on their professional advancement (de Vries, 2005; Vergauwe et al., 2015; Neureiter and Traut-Mattausch, 2016). The prevalence of what is commonly known as “impostor syndromes” among doctoral candidates exacerbates this issue, leading to psychological costs such as fragile self-esteem (Kamarzarin, 2013; Wilkinson et al., 2017), low perceptions of competence (McDowell et al., 2015), negative perfectionism (Ferrari and Thompson, 2006), high anxiety, a sense of lack of control (Vergauwe et al., 2015), negative affectivity, and depression (Wang and Li, 2023).

Impostor syndrome (also commonly called impostor phenomenon and fraud syndrome) is a behavioral phenomenon first described by Pauline Rose Clance as an insecurity in one’s abilities or in achievements, despite any success one has achieved. This is a very common and frustrating phenomenon, where individuals fail to internalize their success and as a result experience feelings of insecurity, anxiety and/or fear of being exposed as fraud in their work, despite verifiable evidence and objectives of one’s success (Kananifar et al., 2015). Individuals most linked to impostor syndrome are high-achieving individuals, disproportionately from academia, particularly in the healthcare field. There is particular interest in studying this phenomenon, as there is an established relationship between Impostor syndrome and other behavioral disorders, including depression, anxiety, and aggravation of other behavioral health problems (Bravata et al., 2020). Although there is no widely accepted medical definition (e.g., the DSM-V criteria), the six original criteria identified by Clance, and expanded upon, can be summarized as a set of characteristics that may or may not be present in an individual with impostor syndrome: perfectionism, super heroism, fear of failure, denial of competence, and success mephophobia (Thomas and Bigatti, 2020).

Crucial to the doctoral journey is mentorship support, with the relationship between the doctoral candidate and supervisor being a pivotal external factor impacting the overall experience (Bravata et al., 2019). Research mentorship, however, faces challenges such as personality mismatches, poor communication of expectations, and misunderstandings, often resulting in ruptures in student-supervisor relationships (Denis and Lison, 2023). Emotional intelligence in supervisors is essential for successful mentorship (Pyhältö et al., 2012), requiring an empathetic approach to adapt to diverse learning styles and understand the emotional states of students (Dugué et al., 2021). Tutor transparency and empathy can go a long way in building confidence and trust in students.

Among emotional skills, empathy is the ability to understand another person’s mood and emotional situation, without recourse to verbal communication (Slimi et al., 2023). Whereby, empathy is a social skill of fundamental importance and represents one of the basic aptitudes for developing emotional competence, especially for fostering social ties, and effective interpersonal communication (Davies, 1990). Empathy is the basis of successful interpersonal relationships, and it is the ability to establish stimulating and inspiring relationships; in addition, the empathic person contributes to their welfare. The concept of empathy has developed through the past century; different studies pointed out different concepts of empathy and its definition (Sánchez-Pérez et al., 2014).

Despite the magnitude of the issue, doctoral program abandonment receives minimal attention from the media, decision-makers, and the scientific community. Empirical research on perseverance in doctoral studies is insufficient, with limited exploration of students’ emotions and the role of supervisors in the emotional process of doctoral candidates (Bošnjaković and Radionov, 2018). Although some studies have alluded to empathic relationships between thesis supervisors and supervisees, particularly in Spanish research (Wollast et al., 2023), few have specifically studied doctoral students’ mental health in relation to their supervisors’ empathic capacity.

This study addresses these gaps by proposing to explore the complex interactions among doctoral candidates, their supervisors, and semiprofessional factors, specifically focusing on impostor syndrome and empathy. By shedding light on psychological challenges in the Tunisian academic context, the research aims to identify potential avenues for psychological intervention and support.

In summary, this study underscores the pressing need to examine the psychological aspects of doctoral studies, emphasizing the impact of impostor syndrome and the crucial role of empathetic mentorship. The investigation contributes to the broader understanding of academia by addressing the well-being of doctoral candidates and promoting interventions to foster a supportive academic environment.

## 2 Materials and methods

### 2.1 Participants

The participant sample consisted of 562 doctoral candidates (*n* = 562), comprising 300 females and 262 males, aged between 25 and 44 years. Among the participants, 244 were employed, while the remaining were students actively seeking employment. Specifically, 110 individuals were in their first year, 122 in the second year, 236 in the third year, 60 in the fourth year, and 34 in the fifth year across five different Tunisian universities, voluntarily participating in this quantitative study. Within this population, 173 females and 178 males were engaged (married, divorced, and/or had children), while the rest were single (Table 1).

**Table 1:** Demographic and professional profile of participating doctoral candidates.

Gender	Marital status	Professional status	Age (years)	Years of thesis enrollment	*N* = 562
Females	Engaged	Unemployed	30.2 ± 3.18	2.05 ± 0.77	116
		Employed	39.4 ± 1.5	4.23 ± 0.92	57
	Single	Unemployed	28.7 ± 3.5	1.67 ± 0.8	114
		Employed	38.08 ± 0.86	3.08 ± 0.28	13
Males	Engaged	Unemployed	34.29 ± 1.6	2.91 ± 0.29	56
		Employed	38.99 ± 1.75	3.42 ± 0.53	122
	Single	Unemployed	29.21 ± 2.62	1.88 ± 0.67	67
		Employed	38.18 ± 1.74	3.29 ± 0.47	17

The sample of mentors (*n* = 152) consisted of 68 females and 84 males, aged between 36 and 59 years. Among them, 43 females and 61 males were engaged (married, divorced, and/or had children), with 95 being experienced (≥10 years) and the rest being novices (less than 10 years) in the field of scientific mentoring (Table 2).

**Table 2:** Demographic and professional profile of participating the supervisor.

Gender	Marital status	Experience (years)	*N* = 152	Age (years)
Females	Engaged	−10	8	37.87 ± 1.80
		10+	35	50.86 ± 4.45
	Single	−10	21	37.38 ± 1.2
		10+	4	44 ± 2.71
Males	Engaged	−10	7	37.57 ± 2.07
		10+	54	53.09 ± 4.32
	Single	−10	21	37.14 ± 1.24
		10+	2	41.5 ± 0.71

### 2.2 Randomization

The study employed a randomized controlled trial design. A statistical data analyst, blinded to the study, facilitated the randomization process. The 562 doctoral candidates (aged 25–44 years) were randomly assigned to either the female (F, *n* = 300) or male (H, *n* = 262) group. Similarly, the 152 mentors (aged 36–59 years) were randomly assigned to either the female (F, *n* = 68) or male (H, *n* = 84) group. The randomization procedure used computer-generated random numbers through JASP software (Jeffreys’s Amazing Statistics Program), version 0.17.3, with essential information stored in a database.

Gender-based randomization was employed to achieve a balanced distribution of participants across different analysis groups while upholding ethical standards and ensuring the reliability of our findings. The primary objective was to minimize potential biases associated with gender distribution among study groups, thereby enhancing the assessment of gender-specific effects on the variables under investigation. Participants were randomly assigned to their respective groups with careful consideration of gender. This ensured that during participant selection and assignment to various study groups (e.g., based on marital or professional status), there was a near-equal representation of male and female participants within each group. Importantly, our approach did not involve strict segregation of male and female groups. All participants, regardless of gender, were included in analyses based on their demographic and professional characteristics as outlined in our study results. Throughout our research, we adhered to ethical guidelines and maintained professional conduct. In summary, gender-based randomization was thoughtfully implemented to enhance the integrity of our findings and ensure equitable representation across study groups.

### 2.3 Design

We hypothesize that there are significant influences on both impostor syndrome and supervisor empathy depending on gender, marital status, professional status, and level of doctoral enrollment. Furthermore, we explored a possible negative correlation between empathy and impostor syndrome. In addition, regarding the relationship between supervisors and students, we conducted a linear regression analysis to examine the relationship between supervisors’ empathy quotient and impostor syndrome among doctoral students. Then, we formulate two hypotheses: (1) there is no significant relationship between supervisors’ empathy quotient and impostor syndrome among doctoral students, null Hypothesis (H₀); (2) there is a significant relationship between supervisors’ empathy quotient and impostor syndrome among doctoral students, alternative Hypothesis (H₁). We conducted a linear regression analysis to examine the relationship between supervisors’ empathy quotient and impostor syndrome among doctoral students.

#### 2.3.1 Impostor phenomenon

The Clance Impostor Phenomenon Scale (CIPS; Clance and Imes, 1978) was employed to measure the impostor syndrome as a trait, as previously validated (Fernández-Fastuca, 2021). Comprising 20 items, the scale assesses various manifestations of the syndrome, including the fear of failure, attribution of success to luck, and rejection of others’ recognition. Scores range from 20 to 100, with higher scores indicating a greater impostor syndrome. Scores ≤40 suggest few impostor syndrome characteristics, 41–60 indicate moderate experiences, 61–80 suggest a high frequency, and scores >80 indicate intense experiences. The Clance Impostor Phenomenon Scale was administered to collect doctoral candidates’ perceptions. Some participants completed questionnaires via email, a validated method for data collection.

#### 2.3.2 Empathy quotient

The empathy quotient of Tunisian supervisors was assessed using the Baron-Cohen and Wheelwright questionnaire (Czerniawski, 2023), and previously validated (Baron-Cohen and Wheelwright, 2004). It is consisting of 60 questions. Respondents could score between 0 and 2 points for each of the 40 questions targeting empathy, with a maximum score of 80.

### 2.4 Statistical analysis

Data were analyzed using JASP software, version 0.17.3. Descriptive statistics were presented as mean ± standard deviation (SD). The Shapiro–Wilk test was conducted to check for normal distribution. An ANOVA was used to compare impostor phenomenon severity among doctoral candidates based on gender, age, marital status, profession, and enrollment year. Additionally, empathy levels among doctoral candidates and supervisors of different ages, marital statuses, and mentoring experiences were compared. Linear correlation was calculated to explore the relationship between impostor syndrome among doctoral candidates and supervisors’ empathy levels.

## 3 Results

### 3.1 Impostor syndrome among doctoral candidates

Our findings demonstrated that female doctoral students experience significantly higher levels of impostor syndrome than their male counterparts [*F*(1, 633) = 45.673, *p* < 0.001, η^2^ = 0.041]. This suggests that gender differences account for 4.1% of the total variance in impostor syndrome.

Marital status shows a statistically significant difference [*F*(1, 633) = 59.969, *p* < 0.001, η^2^ = 0.054], indicating that doctoral students who are married, divorced, or have children exhibit higher levels of impostor syndrome than to single students. The interaction between gender and marital status is not significant [*F*(1, 633) = 0.590, *p* = 0.443, η^2^ = 5.320 × 10^−4^], indicating that the combined effect of gender and marital status is not statistically different from the sum of their individual effects.

Doctoral students who are also employed experience a more pronounced impostor syndrome than those who are not employed [*F*(1, 633) = 136.257, *p* < 0.001, η^2^ = 0.123], suggesting that variations in professional status explain 12.3% of the total variance in impostor syndrome. This means that having a professional activity alongside doctoral studies can exacerbate this feeling of imposture. Employed men (sum of squares = 24,837.26, *F* = 136.26) and employed women both exhibits significantly higher levels of impostor syndrome compared to those who are not employed (*p* < 0.001). Significant interactions include those between marital status and professional status [*F*(1, 633) = 12.789, *p* < 0.001, η^2^ = 0.012], while the triple interaction among gender, marital status, and professional status is marginal [*F*(1, 633) = 3.009, *p* = 0.083, η^2^ = 0.003].

Years of enrollment in the doctoral program were found to have a significant impact on the severity of impostor syndrome [*F*(4, 633) = 125.373, *p* < 0.001, η^2^ = 44.1%], with a notable decrease in syndrome levels as enrollment years advanced, indicating that doctoral students enrolled in their 1st, 2nd, 3rd, 4th, or 5th year show decreasing levels of the syndrome, respectively. The residual variance is significant (115384.855, df = 633), indicating that other factors not included in the model influence impostor syndrome. In summary, the model suggests that gender, marital status, professional status, and thesis enrollment all have significant effects on impostor syndrome. The triple interaction is marginal, indicating that the combined influence of these three factors is at the threshold of statistical significance (Table 3).

**Table 3:** Multifactorial exploration of impostor syndrome determinants through extended ANOVA.

Case	Sum of squares	df	Moyenne des carrés	*F*	*p*	η^2^
Gender	8325.31	1	8325.31	45.67	< 0.001	0.041
Marital status	10931.35	1	10931.35	59.97	< 0.001	0.054
Gender ✻ Marital status	107.51	1	107.51	0.59	0.443	5.32 × 10^−4^
Professional status	24837.26	1	24837.26	136.26	< 0.001	0.123
Gender ✻ Professional status	274.83	1	274.83	1.51	0.220	0.001
Marital status ✻ Professional status	2331.23	1	2331.23	12.79	< 0.001	0.012
Gender ✻ Marital status ✻ Professional status	548.45	1	548.45	3.01	0.083	0.003
Thesis enrollment	89089.45	4	22272.36	125.37	< 0.001	0.441
Residuals	115384.85	633	182.28			

### 3.2 Empathy quotient among supervisors

Supervisors’ empathy plays a crucial role. We investigated the relation between empathy and the gender, marital status and work experience of the supervisor.

Empathy quotient differs significantly by gender, with women (M = 72.54) showing higher levels of empathy than men (M = 65.12). This indicates that gender explains 7.390% of the variance in empathy levels among supervisors.

Similarly, marital status significantly influences empathy quotient [*F*(1) = 46.194, *p* < 0.001, η^2^ = 6.487%], explaining 6.487% of the total variance in empathy levels among supervisors. Married supervisors show higher levels of empathy compared to unmarried supervisors. The interaction effect between gender and marital status is not statistically significant [*F*(1) = 2.966, *p* = 0.087, η^2^ = 0.416%], suggesting that the combined effect of gender and marital status is not statistically different from the sum of their individual effects.

Professional experience significantly impacts empathy quotient [*F*(1) = 27.95, *p* < 0.001, η^2^ = 0.132%], with variations in experience explaining 13.2% of the total variance in empathy levels. Supervisors with more than 10 years of experience show higher levels of empathy than those with less than 10 years of experience (Table 4).

**Table 4:** Exploration of empathy quotient determinants through ANOVA.

Case	Sum of squares	df	Mean squares	*F*	*p*	η^2^
Gender	244772.37	1	244772.37	52.63	< 0.001	7.390
Marital status	214856.41	1	214856.41	46.19	< 0.001	6.487
Gender ✻ Marital status	13795.16	1	13795.16	2.97	0.087	0.416
Work experience	4388.44	1	4388.44	27.95	< 0.001	0.132

### 3.3 Correlation between doctoral impostor syndrome and supervisor empathy

Greater empathy from supervisors is associated with a decrease in impostor syndrome among doctoral students. Table 5 displays the results of a linear regression analysis investigating the impact of empathy quotient on impostor syndrome among doctoral students.

**Table 5:** Linear regression of empathy quotient effects on doctoral impostor syndrome.

Model	Unstandardized	Standard error	Standardized Beta	*T*	*p*
H₀	(Intercept)	73.711	0.821	89.774	< 0.001
H₁	(Intercept)	69.866	2.071	33.743	< 0.001
Empathy quotient	0.122	0.060	0.163	2.019	0.045

Thus, we found a significant negative correlation between supervisors’ empathy quotient and impostor syndrome among doctoral students (*p* = 0.045). This means that we reject the null hypothesis, Ho (no significant relationship or effect between the variables studied), and accept the alternative hypothesis, Hi (there is a significant relationship or effect between the variables studied), indicating that an increase in supervisors’ empathy is indeed associated with a decrease in impostor syndrome.

When the empathy quotient is zero, the impostor syndrome is estimated at 73.711 with a standard error of 0.821 and is highly significant (*p* < 0.001). In H₁ considering a non-zero empathy quotient, yields an intercept estimate of 69.866 with a standard error of 2.071, also highly significant (*p* < 0.001).

Regarding the empathy quotient’s effect, our analysis revealed that an increase in supervisors’ empathy quotient is associated with a decrease in impostor syndrome among doctoral students, with the associated coefficient of 0.122, indicating that a 1-unit increase in the empathy quotient is associated with a decrease of 0.122 units in impostor syndrome.

With a standard error of 0.060, this coefficient presents a standardized correlation (β) of −0.163, signifying a negative correlation. The *T*-value of 2.019 and a *p*-value of 0.045 denote a statistically significant relationship between the empathy quotient and impostor syndrome.

Initially, our study included exploratory elements to identify potential influencing factors. By employing ANOVA and linear regression analyses, we were able to explore both correlational and potential causal relationships between the supervisors’ empathy quotient and impostor syndrome among doctoral students. The results, as presented in Table 5, show a significant negative correlation (*p* = 0.045), leading us to reject the null hypothesis (H₀) and accept the alternative hypothesis (H₁) (Table 5).

## 4 Discussion

The current study explores the correlation between imposter syndrome among doctoral students and the empathy levels of supervisors. The multifactorial analysis reveals substantial connections with gender, marital status, professional status, and thesis enrolment level. The results illuminate the intricate relationship between socio-professional factors, empathy, and imposter syndrome within the academic environment. Poor mental health can have numerous negative consequences for doctoral students and their supervisors, as it can negatively impact quality of life, attrition, and academic productivity. Although the number of doctoral students suffering from psychological problems has increased significantly in recent years, few studies have examined how the supervisor-supervisee relationship may influence the emotional well-being of male and female doctoral students.

Consistent with previous studies that found that women reported statistically significantly higher rates of impostor feelings than men (Cokley et al., 2015; Kananifar et al., 2015; Rajhi et al., 2020), our results demonstrated that female doctoral students experience significantly higher levels of impostor syndrome compared to their male counterparts. This means they feel more negative emotions related to this syndrome. In fact, men and women often deal with their impostor feelings differently (Robertson, 2022). In contrast, other researchers have found no differences in impostor syndrome between men and women (Kananifar et al., 2015; Hutchins and Rainbolt, 2016). In addition, when Brauer and Proyer studied psychology students and professionals, they found gender effects for impostor syndrome only among students, not professionals (Kamarzarrin et al., 2013). Thus, although impostor syndrome is common in women, it also affects men.

Not only do women experience more negative emotions related to this syndrome, but also doctoral students who are married, divorced, or with children also have higher levels of impostor syndrome than single student. This finding conforms to previous work showing that unmarried subjects had higher self-esteem than their peers who are married, divorced, or separated (Brauer and Proyer, 2017).

However, Henning et al. (1998) had shown that married medical students experienced less distress than their single colleagues (Heydari et al., 2008), and it has been suggested that marriage may serve as an antidote to distress.

Another aspect related to impostor syndrome is experience/duty in a position where those with less experience are more likely to experience higher levels of impostor syndrome. For example, the qualitative study by Meister et al. (2014) and Coombs and Fawzy (1982) noted how transitions into leadership roles improve both their responsibilities and visibility to others, often eliciting greater impostor syndrome. Context can impact and even change the direction of IP relationships (Meister et al., 2014).

A key element of this context is the leader–member relationship that “may be the single most powerful connection an employee can build in an organization” (Johns, 2006). Our results indicate a significant correlation between supervisors’ empathy levels and a reduction in imposter syndrome among doctoral students. This discovery highlights the crucial importance of emotional support from supervisors in mitigating the negative impacts of imposter syndrome. The complex interactions between gender, marital status, and professional status underscore the need for tailored support programs considering these socio-professional variables. These results have significant implications for the development of targeted interventions to alleviate imposter syndrome among doctoral students, emphasizing the importance of mentorship programs tailored to gender-specific concerns, marital dynamics, and professional roles.

Furthermore, the conclusion of Nori and Vanttaja (Hui et al., 2004) regarding the link between lack of childhood encouragement and low planning levels during doctoral candidacy align with our own results (Hui et al., 2004). Our analyses confirm these associations, demonstrating a significant correlation between early lack of encouragement and insufficient planning in the doctoral journey. Additionally, previous research has highlighted the relationship between imposter phenomenon and neuroticism (Hui et al., 2004), as well as the impact of self-centered belief of being an imposter on emotional and mental well-being (Kaur and Jain, 2022). Our results confirm these trends, showing a significant positive correlation between neuroticism and imposter syndrome, along with an overuse of emotional regulation strategies associated with poor well-being. On the other hand, the perspectives of Kolontari et al. (2023) and Bonetto et al. (2023) challenge the notion of imposter syndrome and propose a model of affirmation of disability. While our results are consistent with observed manifestations of imposter syndrome, they could benefit from a more in-depth exploration in light of these new perspectives (Bonetto et al., 2023). Statistical analyses could be expanded to examine how these alternative models apply in our specific context.

With respect to doctoral supervision, the importance of considering individual students’ contexts and their relationships to their current and emerging professional identity is clear (Kolontari et al., 2023), as well as psychological safety in student-supervisor relationships (Gunasekera et al., 2021). Our findings confirm the significant effect of psychological safety in reducing impostor syndrome among doctoral students.

Finally, the research of Kong et al. on the intercultural adaptive orientation of advisors, the engagement of student-advisor interactions, and the mediating role of psychological safety provide important insights for understanding interpersonal dynamics in the academic context (Sujon, 2023). Our statistical analyses confirm these mediations, highlighting the importance of intercultural adaptive orientation of advisors and psychological safety in strengthening the academic engagement of international doctoral students.

Our study contributes significantly to the field by highlighting the importance of socio-professional factors and supervisors’ empathy in understanding imposter syndrome. The implications of our findings are crucial for developing targeted support programs aimed at alleviating imposter syndrome among Tunisian doctoral students. For example, implementing mentorship programs tailored to specific concerns related to gender, marital dynamics, and professional roles could enhance doctoral students’ well-being and foster a more inclusive and supportive academic environment.

Our research offers a unique contribution to understanding the factors that exacerbate or alleviate imposter syndrome in the academic context. In conclusion, our results underscore the importance of socio-professional factors and supervisor empathy in comprehending imposter syndrome. These findings open avenues for targeted interventions aimed at improving the well-being of doctoral students, thereby enhancing the academic environment.

## 5 Conclusion

Our in-depth study exploring the correlation between impostor syndrome among doctoral students and their supervisors’ empathy levels reveals significant findings, particularly highlighting the crucial impact of empathy levels.

As a company’s reputation contributes to its sustainable competitive advantage, supervisors’ levels of empathy emerge as a critical element in managing impostor syndrome. The results indicate a significant correlation between supervisors’ levels of empathy and reductions in impostor syndrome among doctoral students. This finding highlights the crucial importance of emotional support from supervisors in mitigating the negative impacts of impostor syndrome.

Expanding this conclusion to the broader context of academic sustainability, it becomes evident that mentoring and supervision programs that promote empathetic communication can play a vital role in improving doctoral student well-being. The positive effect of empathy on the perception of impostor syndrome suggests that interventions focused on developing empathic skills among supervisors could have positive effects on the academic experience of doctoral students.

Empathy is an emotional capacity, which is based on the ability to detect, feel active and share emotions. The supervisor having the skills to emotionally understand how students feel and see things becomes a precursor and inducer of the intellectual skills needed to conduct doctoral research. In interacting with young people, for example, active listening creates a safe and stimulating environment, encouraging them to freely express their logical thinking and scientific imagination. This shows respect and a better understanding of experiences, perspectives, and emotions, which builds trust and self-esteem and ensures the development of a sense of self-esteem and self-confidence (Kong et al., 2023). In addition, the learning environment contributes either positively or negatively to the academic achievements of students (Stojiljković et al., 2012).

Our conclusion highlights an important avenue for future research and initiatives in the field of academic mentoring. Further exploration of how empathy levels can be strengthened and integrated into supervision programs could be a promising path to promoting a healthier academic environment. In conclusion, our findings highlight the importance of socio-professional factors and supervisors’ empathy in understanding impostor syndrome. These findings provide solutions for targeted interventions aimed at improving the well-being of doctoral students, thereby improving the academic environment.

## Data availability statement

The original contributions presented in the study are included in the article/supplementary material; further inquiries can be directed to the corresponding author.

## Ethics statement

The studies involving humans were approved by Institutional Review Board Statement. The study was conducted in accordance with the Declaration of Helsinki for human experimentation. Ethical approval was obtained from the local research ethics committee of the Higher Institute of Sport and Physical Education of Sfax, with reference number 049/2023. The studies were conducted in accordance with the local legislation and institutional requirements. The participants provided their written informed consent to participate in this study. Written informed consent was obtained from the individual(s) for the publication of any potentially identifiable images or data included in this article.

## Author contributions

OS: Writing – review & editing. AM: Conceptualization, Writing – review & editing, Visualization, Supervision. SM: Conceptualization, Writing – review & editing. MB: Writing – original draft.
